# Letter regarding “Clinical features, diagnosis, and survival analysis of dogs with glioma”

**DOI:** 10.1111/jvim.16492

**Published:** 2022-07-27

**Authors:** Carla Rohrer Bley, Valeria Meier, Katrin Beckmann, Frank Steffen

**Affiliations:** ^1^ Division of Radiation Oncology, Vetsuisse Faculty University of Zurich Zurich Switzerland; ^2^ Division of Neurology, Vetsuisse Faculty University of Zurich Zurich Switzerland

Dear Editor,

We have read with interest the article by José‐Lopez and colleagues titled “Clinical features, diagnosis, and survival analysis of dogs with glioma.” In this publication the authors characterize clinicopathologic findings, diagnostic imaging features and survival of a sample of dogs with glioma, along the Comparative Brain Tumor Consortium diagnostic classification.

While we appreciate the thorough investigation of clinicopathological and imaging features, we would like to raise a few points of discussion regarding treatment and survival. Our first concern about the used terminology for “definitive” treatments. The definition of the term “definitive” treatment was based on a study of histiocytic sarcoma involving the CNS^1^ and we believe could be misleading. A systematic review of brain tumor treatment in dogs examines the scientific evidence supporting use of radiation therapy and surgery, and at the same time underlining the lack of evidence for chemotherapy in treatment of brain tumors in dogs.^2^ Since then, no additional findings have been added to the scientific literature on chemotherapy and chemotherapy is not a recommended or acceptable treatment of intracranial tumors at this time.^3^, ^4^ In José‐Lopez et al's study radiation therapy was acknowledged as “anecdotal” treatment. We are surprised about this, quoting Hu et al^2^: “*There has long been a suggestion that combinations of treatment*, *notably surgery and radiotherapy*, *provide the best mode of treatment for brain tumors* (*particularly meningiomas*),(…). *However*, *looking at the data as a whole*, *this conclusion appears inappropriate because the evidence would suggest that adding radiotherapy to surgery has a large impact*,(…) *whereas adding surgery to radiotherapy has no impact*,(…) *suggesting that radiotherapy is the effective modality and surgery may have little additive effect*.” Also, in the meantime, several veterinary publications on outcome after radiotherapy with newer, standard‐of‐care irradiation devices have become available in the peer‐reviewed literature.^5^, ^6^, ^7^ In José‐Lopez et al's study, treatments seem chosen and distributed randomly, without information on dosages, dose intensity or quality of radiation therapy. This leads to a substantial lack of quality validation or standardization of the claimed “definitive” treatments and in our view could mislead the uncritical or inexperienced reader and client in regard to possible outcome of dogs with glial tumors. Such variable treatments should also not be used to make predictions on prognostic indicators.

Surgery indeed often leads to subpar outcome likely because a high percentage of tumors are not amenable to an oncological definitive (clean) resection in this often highly infiltrative disease. Cytoreductive surgeries/debulking in oncology cannot be considered a stand‐alone therapy for any type of tumor and in general serve only to palliate. Median survival time after surgery—often anecdotally claimed as the standard treatment by neurologists and surgeons—remains unfortunately not well described and is at best short, around 6 months.^8^

Radiation oncologists have shown various times that dogs with glioma have an excellent outcome after radiation therapy, when compared to symptomatic, palliative treatment. Using the state‐of‐the‐art irradiation devices of the last decade, time to progression has oscillated around 18 months, with disease‐specific survivals around 20 months.^5^, ^6^, ^7^ These dogs have a good life after treatment, even though tumors might subsequently recur or disseminate within the CNS.

Of second concern is the that >50% of the 91 dogs were immediately euthanized upon diagnosis, which was based on results of imaging. While these dogs were excluded in the survival analysis, recommendations with regard to euthanasia or treatment were most likely made based on initial first diagnostic imaging. Making *treatment* decisions based on diagnostic imaging only is often criticized, (own experience). Noninvasive diagnosis, which is based on imaging, is often chosen by clinical radiation oncologists and neurologists because of the perceived risk of biopsy in dogs with this disease. Furthermore, it does not appear to be consistent to criticize *treatment‐decision‐making* on imaging diagnosis, but then recommending *euthanasia* on the other hand directly after diagnostic imaging.

Our third concern is the “main takeaway” from José‐Lopez et al that no associations were found between clinicopathologic findings or survival and tumor type or grade. Unfortunately, in spite of the meticulous description of clinical and diagnostic imaging features, the tumor volume, one of the only factors so far found to possibly be of relevance with regard to outcome,^5^, ^7^ was not included in the evaluation.

It is surprising to us that none of the described prognostic variables were of prognostic value. This would render all the recommendations for pretreatment biopsies or advanced imaging baseless, as extensive diagnostics clinically only serve to refine the guide to treatment‐decision making. It would also disqualify the valuable findings of this study, such as the description of margins, MR intensities, ventricular contact and imaging association with presumed histologic classification. We believe this perceived irrelevance of histopathological classification and imaging findings as prognostic factors could have been masked by the use of “definitive” treatments that were not, in fact, definitive.

We thank Jose‐Lopez et al for their contribution and we recommend continuing to expand our knowledge about diagnostics, optimal treatment and prognostic factors in dogs with glioma.

Respectfully,
